# The assessment and management of diabetes related lower limb problems in India-an action research approach to integrating best practice

**DOI:** 10.1186/1757-1146-7-30

**Published:** 2014-05-18

**Authors:** Michael Harrison-Blount, Michelle Cullen, Christopher J Nester, Anita E Williams

**Affiliations:** 1 Directorate of Podiatry and Prosthetics & Orthotics, School of Health Sciences, College of health and social care, University of Salford, Frederick Road, Salford M6 6PU, UK

**Keywords:** Diabetes, Focus groups, Participatory action research, Care pathway, Foot screening

## Abstract

**Background:**

In this article the authors explore the current issues and barriers related to achieving successful outcomes to diabetic foot complications in India. This was achieved by engaging clinicians in taking ownership of the problems and facilitating them in the identification of solutions to action change in clinical practice.

**Methods**

This was accomplished through facilitating participants in this study via a process of problem identification and planning, the first phases of an action research cycle approach. The methods of data collection were focus groups, observations and individual conversations. The data were analysed using a thematic framework.

**Results**

Based on the practitioner’s experiences and opinions, key themes were identified. These themes had the potential to inform the changes needed in clinical practice, to overcome barriers and embed ownership of the solutions. Five themes were identified highlighting: concerns over a fragmented service; local recognition of need; lack of standardised care pathways; lack of structured assessment and an absence of annual foot screening. Combined, the issues identified were thought to be important in preventing timely assessment and management of foot problems.

**Conclusion**

It was unanimously agreed that a formalised process of foot assessment should be developed and implemented as part of the subsequent phases of the action research process, which the authors intended to take forward and report in a further paper. The aim of which is to guide triage, education, care pathways, audit and evaluation of outcomes. Facilitation of the clinicians in developing a program and screening tool to implement and teach these skills to others could be an important step in reducing the number of high-risk cases that are often resulting in the amputation of limbs.

## Background

India has the highest prevalence of people with diabetes in the world [1,2] which is predicted to increase to 120.9 million by 2030 [3]. Aligned with this is an ‘epidemic’ of diabetic foot complications with ulceration and infections having devastating consequences for lower limb morbidity [4]. With trivial and avoidable foot lesions known to precede 85% of leg amputations [5], the World Diabetes Federation asserts that amputation rates can be reduced by 49-85% if strategies for preventing and treating foot lesions are implemented [4]. A multidisciplinary approach with preventative strategies can reduce amputation rates by more than 50% [6].

Evidence from the Western context [5,7-17] provides clear guidance for effective foot health assessment and management. It would be natural to consider a transfer of this ‘best practice’ from the West as being a potential and speedy solution to reducing the incidence and prevalence of diabetic foot problems in India. However, it is important to recognise that this best practice was born out of a Western health care system and as a result, may not transfer seamlessly to a system which is culturally very different [9]. The social, economic and cultural profile of patients in India, their specific foot problems, the professional disciplines they present to, the clinical locations, resources and communication management are all factors which have the potential to block the transfer of Western best practice into India. Certainly, simply saying to colleagues in India ‘here is what we do, now get on with it’ is unlikely to deliver lasting and effective changes in practice. Instead, understanding the philosophy and delivery of foot health management in the Indian context is a crucial step in supporting the changes in clinical practice and hence the negative outcomes of diabetic foot complications. Previous attempts to transfer Western foot health practice to India did not report this crucial step and this puts at risk any short term benefits they may have generated, [1,9,18].

Achieving sustained change in health care practice will inevitably rely on lasting engagement of the practitioners within that service [19]. It is widely recognised that implementing change into clinical practice is difficult even when clinicians agree with the evidence that demonstrates a need for change [20-26]. Approaches that can further engage practitioners in the change process and that build local ownership of solutions are likely to offer a better chance of a lasting change in practice. Action research [27] is particularly suited to this challenge because it has the potential to enhance transfer of knowledge whilst concurrently addressing the barriers to change that exist, because of differences in health care context between the West and India. In the context of foot health in diabetes, it has the potential to be a vehicle for identifying the multiple and complex differences between Western and Indian contexts providing local ownership of the potential solutions and change process.

The aim of this research therefore was to better understand the local (Indian) context related to the assessment and management of diabetes related foot health, to engage clinicians in taking ownership of the problem, to support the identification of locally owned solutions to the problems and then action change in clinical practice. The purpose was to enhance the likelihood of local ownership of the transfer of Western practice to the Indian context and increase the chances of lasting change at the specific health care setting.

## Subjects, materials and methods

Approval for the study was obtained from the University of Salford ethics committee and the hospital governance team at a major university hospital situated in Chennai, India. The problem identification and planning phases of the action research approach were adopted to facilitate a mutually collaborative interpretivist perspective between the researcher and the clinicians [19,28]. Achieving this synergy is a crucial element to facilitating change, which is sustained over the longer term [29]. Action research is an iterative cyclical process, which involves a reflective cycle of problem identification, action planning, action, reflection, evaluation and replanning [30,31]. The focus of this research was on the initial phases of the action research cycle. In order to achieve these elements, focus groups, observations and individual conversations were the methods through which data was collected. The triangulation that the use of these multiple methods allowed aimed to ensure that the data was as reliable as possible and reflected fully what was being investigated. The process of action research was aligned with the protocol and with justification for each stage (Table 1).

**Table 1:** Research protocol as aligned with the action research process

**Phase of action research**	**Methodology**	**Aim**
1) Ownership	Observations and informal discussions with hospital staff.	To inform content of focus group question template.
		To capture an untainted picture of current practice before the formal focus groups.
2) Problem identification	Focus group with recruited participants from hospital staff.	Capitalises’ on the interaction between and among participants to stimulate and refine thoughts and perspectives’ (18).
		Useful in deriving collective opinions of groups.
		Useful when there are power differences between the decision-makers and/or professionals, when exploring the degree of consensus on a given topic (19).
3) Initial Action planning	Observations and informal discussions following the focus group.	To observe and discuss further opinions regarding pertinent points identified in the focus group.
		To allow staff to speak more about the points discussed free from barriers such as seniority, gender, caste and internal politics.
		To clarify the points raised and view them in action.
		To facilitate the exploration of ideas around change.

Data collection was carried out in two phases. Phase 1 was exploratory where the researchers gained insight into the context through informal discussions with the practitioners. This was aligned with general observations focusing on aspects of service delivery and management. This phase allowed the researcher and hospital staff to co-create a set of open questions to help facilitate the focus group in Phase 2 and ensured it reflected the Indian context (Table 2).

**Table 2:** Question Template used to facilitate the focus group

	
INTRODUCTORY QUESTION	Please can you describe the current patient demographics of the hospital?
TRANSITION QUESTION	What types of assessment forms are currently being used and can you give examples of these throughout the hospital?
KEY QUESTION	Can you describe your thoughts on the current assessment and triage process? Who is responsible for doing this?
	Does the process currently work?
	Can you give some examples of the positives and negative aspects of this process?
KEY QUESTION	What details would you want to capture from a foot assessment tool?
KEY QUESTION	What do you perceive a successful assessment tool would achieve?
ENDING QUESTION	If you had the opportunity to change it how would you see structured assessment and triage working?
FINAL QUESTION	Is there anything else that anyone feels we should have talked about but didn’t?

In Phase 2, participants were then purposively recruited for the focus group from a variety of speciality departments observed in Phase 1. The inclusion criteria for the participants were that they must be doctors or health care professionals (HCPs) in positions as heads or assistant heads of a department, and/or they were practitioners who were regularly assessing and treating patients with foot health problems.

Eleven members of staff who met these criteria were invited to join and verbal and written consent was gained at the focus group session. Nine members of clinical staff from the hospital participated in the focus group; two additional members of staff could not due to clinical commitments (Table 3). The focus group discussion was recorded using a digital voice recorder.

**Table 3:** List of invited participants and corresponding code to ensure anonymity

**Participant (P) code**	**Role**
P1	Consultant General Physician
P2	Consultant General Physician
P3	Consultant Dermatologist
P4	Orthopaedic surgeon (Lead member of staff, key contact)
P5	General surgeon (Head of service)
P6	Consultant Sports Medicine
P7	Consultant Diabetologist (Lead member of staff, key contact)
P8	Head of Physiotherapy
P9	Head orthotist
P10	Consultant Vascular Surgeon (Head of service/Unable to attend)
P11	Consultant endocrinologist (Head of service/unable to attend)

Following data collection the dialogue was transcribed verbatim by the researcher and codes were assigned to participants (Participant 1 - P1 and so on) to ensure confidentiality. A co-researcher made field notes and these observations supplemented the data from the focus group. Additional observations and informal conversations then further explored themes that emerged from the focus group, to add rigor to results and the opportunity for participants to voice opinions around possible solutions. The implementation and evaluation of which would form the subsequent phases of the action research process to complete the cycle. Two lead members of staff from the hospital had been identified prior to the research process as the key contacts. In addition they had been given the task to take forward and implement any of the actions beyond phase 3, alongside the researcher. They were purposively selected to verify the results as not only were they members of the focus group but also they had the power to be the change agents.

A thematic framework approach was used to analyse the data [32]. This process allows codes and themes to be developed and defined, focusing on both the content and meaning of the data. This iterative framework analysis was employed [33-35] with initial familiarisation of transcribed data both in audio and written format. This gave a sense of emerging themes together with the additional data obtained from the observations, conversations and field notes.

## Results

Nine initial sub themes were derived from the transcribed data collected in phase 2. These initial sub themes were then synthesised into five themes to include the data collected in the field notes during the observations and informal discussions (Table 4).

**Table 4:** Theme development

**Sub-Themes**		**Main Themes**
1	Demographics	*Theme 1- Local definition and recognition of need* describes the patient demographic and types of foot conditions the hospital and individual departments are dealing with day to day. Including how these problems are currently managed and the feelings of the professional group in relation to the points identified.
2	Common Conditions	
3	Current Practice	
4	Assessment	*Theme 2 - Process of current foot assessment* describes how the hospital currently assesses foot health.
5	Referral	*Theme 3 – Barriers to current diabetic foot care* highlights the operational structure and the existing problems within current practice that hinder the standardisation of foot assessment and management.
6	Resources	
3a	Current Practice	
7	Content	*Theme 4 – Content of assessment* describes what the participants believe would broadly make up the content of a foot health assessment tool.
8	Outcomes	*Theme 5 - Desired Outcomes of foot assessment and the opportunity to change* describes what a successful foot health assessment could achieve and also the desire to make changes and integrate them into current practice.
9	Change	

### Theme 1- Local definition and recognition of need

It was reported that patients travel from a wide area (approximately 600 sq miles) to access services and medication as these are free or subsidised. Medical records are kept in the patient’s possession rather than at the hospital. To receive medication they must have the records with them and also attend for the general medical checks subsequent to prescription.

Foot health care is provided by a diversity of departments and hence the extent of foot health assessment is dependent on the practices of those individual departments. In some cases a department may not do any routine screening assessment unless the patients report a problem. Some participants were keen to insist that assessment was routine for the diabetic population as P2 explains;

*“We will also monitor them for diabetic foot complications we can do a screening test for them and assess them periodically and try and pick up early what problems they have. We can address this and then prevent them from getting a problem, foot problems like amputation”.* P2 (general physician)

However this was in the context of their own department.

Aligned with the responses in the focus group, observations made by the researchers in the diabetes outpatient clinic during Phase 1 revealed that foot examination was only carried out if the patient complained of a problem or a condition was visibly evident, such as ulceration. Precursors to foot ulceration such as neurological status, alterations in foot structure, nail pathology, or callus were not assessed. When an examination did take place it was not systematic or structured and was superficial, often being a limited visual inspection lasting around 30 seconds per foot followed occasionally by a subjective palpation of pulses.

Statements about the foot health of the population revealed that many conditions exist and are not all associated with diabetes. Three participants in the group were keen to share their experiences of the conditions they are regularly confronting;

*“Dermatology directly will be more of leprosy and smaller problems,* P3 speaks at same time *“…callosities, trophic ulcers, leprosy and plantar warts.” But major wounds with loss of toe or half the toe nail missing they will go to general surgeons”*. P1 (general physician)

*“We see a lot of loss of sensation and perception we have had a few examples of patients who are fine with everything but have 100% reduction in one foot sensation that altered them the whole life, their issues are entirely different”.* P8 (Physiotherapist)

Conversations with practitioners in the vascular department during Phase 1 revealed that in relation to diabetes the majority of lesions were a result of neuropathy, with few being associated with vascular insufficiency. Buergers disease was a significant problem because of the high prevalence of smoking and use of homemade cigarettes using raw tobacco. WHO estimated a prevalence of tobacco consumption in India of all forms at 65% and 33%, respectively, among men and women, based on small scale studies conducted in the past compared to 21% in the UK [36-39]. The prevalence of the disease in India among all patients with peripheral arterial disease is as high as 45 to 63% [40] and this contributed to a large percentage of patients in vascular surgery.

*“Surgery is difficult on the small vessels involved and patients who had successful treatment often had a reoccurrence of Beurgers disease because of continued smoking”*. P10 (vascular surgeon).

The scale and variety of foot problems invoked emotions such as frustration and the sense of being overwhelmed. Observation of general surgery in Phase 1 indicated a significant number of foot wounds were referred from other disciplines, into a single department without triage.

### Theme 2 - Current foot assessment

The participants discussed current assessment procedures and identified that there was nothing specifically designed for foot assessment

*“A lot of forms are used but nothing to look just at the foot conditions. Things will be written down in the patient’s notes if they are important. Some areas will have some things to record about feet like the vascular department when they are looking at pulses and things. But just written in the notes really”*. P2 (general physician)

The group unanimously agreed that this was the current practice. It was apparent that no risk categorisation was used to highlight at risk patients, nor is foot assessment or triage routinely carried out for all diabetic patients. Important foot conditions were highlighted as ulceration and/or infection, with no recognition given to those factors that can act as precursors. It appears that most of the time the patient stays under the care of the first clinician they see and there was no evidence of cross specialty referral and thus no care pathways. An example is given from dermatology;

*“The person who sees the patient first, he takes care of the patient, he first classes the patient and follows the patient, he takes care of them, that is the thing, we don’t have a system we don’t have an assessment chart that we have devised”*. P3 (dermatologist)

It is obvious that an awareness exists that these assessments should be done routinely, and that individual departments are receiving large numbers of referrals for major foot problems, rather than identifying solutions to prevent them. No systematic assessment, triage or collaborative approaches across the hospital departments exists. Although there is recognition that introduction of this might enable care to be tailored to patients specific needs.

### Theme 3 - Current barriers to delivering diabetic foot care

It became apparent that early detection of complications was difficult to achieve because of the lack of centralised care or a dedicated team for diabetic foot assessment. This resulted in occasional and non specific referrals, limited teamwork and inadequate monitoring of patients, as P7 reveals

*“So that it goes into that branch and from then they have the primary care of the patient but other people should still be involved. At the moment it gets segregated and patients don’t come back”*. P7 (Diabetologist)

Currently the patient decides which department they are going to attend. For foot related problems this can be variable because of lack of information, minimal patient education and no designated foot clinic. The first physician they see, often the general physician, will then make a decision on whether a referral to another speciality is required. The general physicians have a clear idea of the importance of early referral and their role in centralised assessment, for example;

*“Sometimes they come to the general physicians for their diabetes…they also say that this problem is not solved in the foot that is when we say yes you need dressing and some debridement, go to the general surgeon or if it’s for the dermatology department they go to dermatology”*. P1 (general physician)

Observations revealed that the majority of consultations in endocrinology were to review blood sugar levels, give advice on diet, and take blood pressure measurements. Routine examination of the feet was not undertaken unless the patient highlighted a foot problem. We observed a range of simple foot lesions, but the medical staff did not have the time to routinely check feet due to the high volume of patients attending these clinics. Three doctors saw approximately ninety patients in a two hour session. Although the question was occasionally asked ‘how are your feet’, ‘No problems’ was often the answer. It was observed that advice was given if patients were known to be carrying out unsafe self-care such as the use of non-sterile razor blades for hard skin removal. This often consisted of the phrase “you should not be doing that” and no alternative approaches or explanation of risk was offered.

There was a unanimous understanding by the participants, and those with whom conversations were held, that patients with feet at risk of serious complications were being identified too late. Agreement was also made that assessment should be done by a physician and not a supporting member of staff. Once the physician sees the patient parts of the assessment may then be delegated e.g. foot pulses/monofilament. This assumes that the correct referral happens as the physicians themselves have done the majority of the assessment. Little responsibility is given to the support staff to carry out assessments and make the correct referral decisions.

### Theme 4 - Content of assessment

It was unanimously considered that if an assessment tool was developed and implemented in the further stages of the action research cycle, then this should not be limited to those patients with diabetes related foot problems. This indicates that ignoring foot problems is a widespread issue across all medical specialities. It was made clear that medical and medication history should be recorded upon any devised assessment, to fully inform the practitioner who receives the referral, of the patient’s systemic health and not just information specific to the foot complaint.

*“Even the assessment tool should also have a column for systemic diseases which he has got, diabetes, immuno-compromised. Sometimes most of these assessment tools can be very limited to what it has got, for example it does not take into consideration things like underlying diabetes or if he is on any medications, so all these things will also give us some clue”.* P3 (dermatologist)

In addition to this P2 commented

*“Our expectations of an assessment tool are it should be simple, not too complicated and easily reproducible”.* P2 (general physician)

The overall opinion from the group was that a possible solution to the problems identified would be the development and introduction of an assessment tool, which would be seen positively and embraced in practice. It was clear however that it needed to be holistic and tailored to the context in which it would be used.

### Theme 5 - Desired outcomes from foot health assessment

Several of the participants suggested that a foot health assessment tool would allow for audit, evaluation of outcomes and to measure potential improvements.

*“The role of assessment is to protect/detect their early trivial lesions to prevent against later complications and a tool should be easily used in the follow up visits. Also so that we will know if there is an improvement. It should also allow us to know if the patient again has to be investigated so that when we follow up the patient with the tool it will alert us”.* P3 (dermatologist)

*“We would easily be able to compare what he was, what he is now and whether we have done a good job”*. P1 (general physician)

It was also considered, that although one practitioner would be responsible for the patient the wider team should still be involved

*“The assessment should naturally lead to the different specialties”*. P7 (endocrinologist)

## Discussion

Previous studies [1,9,41] have not explored in depth the obstacles that exist to transferring foot health care knowledge and practice in India. The development of a set of open questions acted as a useful guide to structure the subsequent focus group and aligned with the action research philosophy of participatory engagement. Research has suggested that the planning of questions is useful in focus groups involving people from culturally and linguistically diverse backgrounds. In particular, they indicate that questions that were too open failed to yield answers sufficiently detailed to be meaningful [42]. The problem identification and planning phases of action research reported here have facilitated a period of enquiry, which describes, interprets and explains social situations in the setting where change is required. Facilitation of the action research approach has provided the researchers and participants with the tools to complete the subsequent action and reflection phases of the cycle in a further study. Completion of this cycle will facilitate a change intervention aimed at involvement of practitioners and improvement in practice [43,44]. It is problem focused, context specific, and future orientated [19,27]. In contrast to previous work [1,9,18], our action research approach is unique in this context and has provided a vehicle for these practitioners to reveal personal accounts of the organisational structure, including current protocols and procedures. It has also offered the opportunity for them to identify, current issues, possible barriers, potential solutions, concerns for the future and most importantly opportunities for change. A strikingly obvious enthusiasm and desire to improve foot health practice existed amongst the participants. This was evidenced through an approach where the participants identified the potential for developing foot health assessment, with the aim of improving the outcomes of diabetes related foot complications and pathology.

Focus groups have been a useful method for both data collection and to allow the participants to reflect and provide the information and the consensus opinion required to underpin change in the clinical context to better meet local needs [45]. As seen from the observations and the personal accounts of the participants in this study, the variety of problems with foot health differed very little from those evidenced in the West. However, both the scale and severity of ulceration and infection are resulting in higher rates of amputation, with an estimated 50,000 amputations occurring every year as a result of diabetes related foot problems [3]. This equates to an amputation rate of 45% for diabetic foot problems alone. This aligns with previous work indicating that areas where foot protection guidelines are not routinely implemented have a greater incidence of severe foot health problems [1,4,5,41]. Furthermore through observation and discussion in phase 1 it was identified early that follow up treatment was carried out at the hospital and that the outcomes of foot health interventions were rarely measured. The participants recognised this problem both locally and nationally and had concerns about how the increased incidence and prevalence would impact on their service. However, this awareness and sense of urgency was not mirrored by action to address the current problems, let alone strategic planning for the future demands on the service. Hence, a fragmented service exists with no integrated multidisciplinary approach, no care pathways [18] no structured assessment tool, no annual foot screening [15] and obstacles preventing timely assessment and management. The participants expressed frustration at all of these issues, recognising that patients present too late for any of these to be of benefit. All these factors have resulted in a ‘fire fighting’ approach with the focus being the treatment of the presenting foot problem rather than developing the therapeutic relationship that underpins education, negotiation and positive health behaviour [46].

Our approach has enabled us to better understand a series of steps required to transfer best practices into the local Indian context. It was very clear that a fundamental aspect to changing the current situation would be training in effective consultation and assessment skills so that the patient becomes the focus. There is evidence to support the effectiveness of these being taught in other areas [47]. The lack of opportunity for the patient or time put aside for an assessment to take place all reinforced the impact of the problem. Further to this was the lack of diabetic foot education occurring during consultations in relation to prevention and self-identification of risk. Whether the participants all had this knowledge to allow them to educate the patients is not known but it does indicate a lack of acknowledgement from the practitioners about the impact of identifying simple lesions and educating the patients on the importance of self-monitoring. This lack of communication evident between practitioner and patient detracts from the development of a therapeutic relationship whereby discussions and negotiations can support greater understanding for both. Clearly the beginning of this process is the identification of the potential risk to be discussed and that prevention and early detection are precipitated by communication of these risks to the patient. It is well known that timely identification of trivial lesions and structured assessment are successful in managing the diabetic foot [16]. Subsequent phases would then allow the development and implementation of a programme where patients are educated to identify trivial foot lesions, attend in a timely fashion and then receive early foot screening using a collaboratively designed assessment tool to guide these skills, could be an important step in reducing the number of high-risk cases that are often resulting in the amputation of limbs.

Furthermore, it is considered that this training should be done within a multidisciplinary foot care team who have the competencies to deliver care for patients with diabetic foot problems [16].

The study was not without its limitations. It could be argued that certain research methods are not culturally suitable and researchers may unconsciously interpret experiences of other ‘cultures’ through the lens of their own cultural beliefs and values. This could potentially lead to ethnocentric assumptions that are inaccurate and incomplete. Action research has given the participants the opportunity to facilitate and offer support in identifying the problems through a formal process and guiding them in the development of action plans. The focus group also ensured the exploration, validation and clarification of their perspectives and beliefs despite the reticence that is known to stifle discussion [45]. The hierarchical structure within the hospital was evident in relation to both professional role and caste with those deemed of less importance not speaking until others had spoken. While respecting this, the researcher ensured that all had the opportunity to speak during the focus groups. The informal interviews and observations were used to capture the non-verbal nuances and provide clarity as to the appropriate interpretation of data from the group work.

Future research will allow the authors to complete the action research cycle begun here. Through the phases of action and implementation the authors seek to facilitate the participants to develop a context specific assessment tool to aid identification of foot problems, aligned with practitioner training and guidelines to support the process. This will then provide an evidenced based assessment tool and the practitioners with the skill to use it to identify risk, engage in foot health education and collect prevalence data. This information can then be used to plan treatments for individual patients and plan for future services for this patient group. In addition, the engagement of key decision makers created ownership, leadership and a momentum for change in the exploration of developing a dedicated foot clinic where a foot health assessment tool could be piloted and implemented. This clinic could become a “hub” for foot health knowledge, education, training and development of practices effective in the local context, steps critical to reducing the number of cases that result in the amputation of limbs. The action research approach process has thus enabled understanding not only of what needs to be done, but also the “how to do it”.

Agreement was achieved that in the next phases of action and implementation the researchers would continue to take a practitioner focused approach, to allow for ownership in the development of a screening/assessment tool. Working collaboratively in this respect should ensure that it incorporates current best practice guidelines, is culturally sensitive and that ownership is taken by those who are going to use it. Implementation of the assessment tool will be supplemented by support, training and written guidelines.

## Conclusion

This facilitation of problem identification and planning to identify the need for change has given the participants the momentum to drive change forward and to plan improvements to services they deliver. This approach has provided us with a better understanding of the local Indian context and more importantly given ownership of the problem to the participants. There was clear recognition from all participants that a means of assessment, risk classification and alignment with treatment guidelines was required. Local ownership of the problem and engagement in the process of change has also been achieved [32] increasing the likelihood of sustainable and long lasting benefits. The result of facilitating the first phases of the action research cycle has created a legacy, which in practical terms will be the development of a foot health assessment tool which addresses local need and builds on Western best practice, but in a way that is sensitive of the Indian context.

## Competing interests

The authors declared no potential conflicts of interest with respect to the research, authorship, and/or publication of this article.

## Authors’ contributions

MHB Facilitated and led the focus groups, Transcribed and analysed the data and drafted the manuscript. MC acted as research assistant during the focus groups and contributed towards the literature review. AW and CN acted as PhD supervisors for MHB. All authors read and approved the final manuscript.
